# Statistical signals of copying are robust to time- and space-averaging

**DOI:** 10.1017/ehs.2023.5

**Published:** 2023-04-13

**Authors:** Mason Youngblood, Helena Miton, Olivier Morin

**Affiliations:** 1 Minds and Traditions Group, Max Planck Institute for Geoanthropology, Jena, Germany; 2 Santa Fe Institute, Santa Fe, New Mexico, USA; 3 Institut Jean Nicod, ENS, EHESS, PSL University, CNRS, Paris, France

**Keywords:** copying, complexity, time-averaging, space-averaging, generative inference

## Abstract

Cattle brands (ownership marks left on animals) are subject to forces influencing other graphic codes: the copying of constituent parts, pressure for distinctiveness and pressure for complexity. The historical record of cattle brands in some US states is complete owing to legal registration, providing a unique opportunity to assess how sampling processes leading to time- and space-averaging influence our ability to make inferences from limited datasets in fields like archaeology. In this preregistered study, we used a dataset of ~81,000 Kansas cattle brands (1990–2016) to explore two aspects: (1) the relative influence of copying, pressure for distinctiveness and pressure for complexity on the creation and diffusion of brand components; and (2) the effects of time- and space-averaging on statistical signals. By conducting generative inference with an agent-based model, we found that the patterns in our data are consistent with copying and pressure for intermediate complexity. In addition, by comparing mixed and structured datasets, we found that these statistical signals of copying are robust to, and possibly boosted by, time- and space-averaging.

## Graphical abstract

**Figure:**
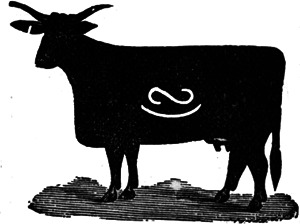

**Social media summary:** Cattle brands provide clues about how time- and space-averaging might affect statistical signals in archaeological data.

## 1. Introduction

**Figure 1.**
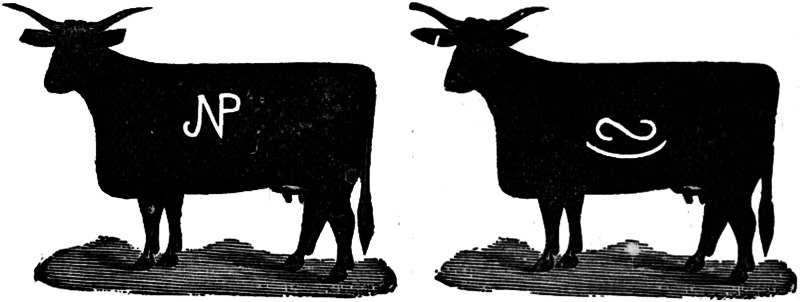
Examples of two cattle brands registered in the US state of Kansas in 1884 (https://www.kansasmemory.org/item/309140/). Both brands are composed of two components: on the left a stylised ‘N’ and ‘P’, and on the right a rotated ‘(‘ and ‘S’).

Cattle branding, or the use of hot irons to leave marks on animals, is a common practice that dates back to at least ~1500 BCE (Insoll et al., 2015; van der Moezel, 2016; Wolfenstine, 1970). Cattle brands are emblems – simple graphic codes that do not encode language (Morin et al., 2020). In North America, cattle brands were introduced by the Spanish in the sixteenth century (Brand, 1961) and are applied to one part of the animals’ body to denote ownership and deter theft. Today, cattle branding in most parts of the USA is regulated by state organisations that register new brands and ensure that they are unique. Cattle brands in the USA are usually composed of two to four components – constituent parts that include letters, numbers and symbols (Figure 1). Letters often correspond to initials or ranch names, but sometimes the reverse is true and ranches (and even towns) are named after cattle brands (Lombard & Du Plessis, 2016, 2019). The symbols in brands are more arbitrary and include everything from punctuation marks to state outlines and animals.

In any symbolic system used for communication, including simple emblems like cattle brands, there needs to be a way to distinguish symbols from one another. If more distinctive symbols are less likely to be confused with other symbols, then pressure for distinctiveness should cause symbolic systems to become more effective over time. In the evolution of writing and speech, for example, pressure for distinctiveness is thought to have been fundamental in maximising the signal space for better expressivity (de Boer, 2005, 2015; Flemming, 2002; Liljencrants & Lindblom, 1972; Smith & Kirby, 2008). Distinctiveness is probably an important consideration when ranchers create new cattle brands, since differentiating animals from one another is their primary function (Gracy II, 2016). Distinctiveness is interesting to us chiefly because it cannot be maximised without countering two important mechanisms that also shape emblems: copying and pressure for reduced complexity.

When graphic elements are copied the resulting emblems are less distinctive than they would have been if they were independently created. Successful graphic forms, when they spread to new users, tend to become less informative as a result. The designs that ancient Greek city-states minted on their coins were often adopted by entirely different cities. When this happened, the amount of information that coins carried about the cities that minted them declined: the same emblem became a poorer indicator of its city of origin (Pavlek et al., 2019). Likewise, the copying of heraldic coats of arms in medieval Europe decreased the distinctiveness of copied arms (Morin & Miton, 2018).

Pressure for reduced complexity, which occurs because simpler symbols are easier to perceive, learn and produce (Miton & Morin, 2021; Smith, 2020), can also reduce the distinctiveness of emblems. One possible definition of complexity is the number of ways that a shape could vary from its actual form (van der Helm, 2014). Intuitively, there are relatively few ways to transform the letter ‘O’ into another letter without adding to it, whereas a character like 尴 has many more degrees of freedom. Complex shapes are more likely to be distinctive because they can vary along a greater number of dimensions (Miton & Morin, 2021). This notion predicts (among other things) that written letters should be more complex in larger scripts (e.g. Devanagari vs. Greek), a phenomenon that has been shown using a variety of metrics for visual complexity (Chang et al., 2018; Miton & Morin, 2021). Graphic codes generally evolve to have reduced complexity over time (Kelly et al., 2021; Tamariz & Kirby, 2015), although this tendency is not universal (Miton & Morin, 2019; Tran et al., 2021).

### 1.1. First aim: distinctiveness, copying and complexity

Variation in cattle brand designs is also likely to be shaped by pressure for distinctiveness, copying and complexity, but the relative importance of these forces is unknown. We make a distinction between distinctiveness (outcome at the level of brands) and *pressure for* distinctiveness (process at the level of components). In our model, pressure for distinctiveness occurs when the rarest components have the highest probability of being included in brand designs. This component-level process is one of several factors (including complexity, as described above) that affect the resulting distinctiveness of brands.

State regulations forbid the exact copying of another rancher's brand: each ranch must register a brand that is unique in one way or another – meaning that its component symbols, the way they are rotated or arranged, their location on the animal, or all of the above, are unique.^1^ However, they do not forbid the copying of components or even sets of components, as long as the result is not an identical replication of another brand. Ranchers may copy components out of convenience from published brand books, use common components because of standardised design or reading conventions (e.g. from branding manuals), or modify existing brands with new components (e.g. as observed among pastoralist groups and cattle thieves) (Arnold, 1965; Landais, 2001; Thomas, 1967; Wolfenstine, 1970). Among East African pastoralists, the copying of brands within male lineages is thought to explain the high level of sharing between groups, and possibly even the occurrence of contemporary brands in rock art in the area (Lynch & Robbins, 1977). Other aspects of cattle branding, such as the branding rituals themselves, are also known to be copied (Bird Rondeau, 2013).

As for complexity, operationalised here as the number of components in a brand, the peculiar technology of cattle branding imposes constraints of its own. In the context of cattle branding, designs that are too complex may be more difficult to physically blacksmith, more difficult to read from a distance, and more painful for the animal (Newman, 2007; Stamp, 2013). On the other hand, pressure for reduced complexity in cattle brands is probably limited by the fact that simpler brands are more easily modified by cattle thieves (Adams, 1906; Gracy II, 2016). Cattle theft remains a significant problem (Keen, 2013), and while other technologies such as radio-frequency identification and ear-tagging are less painful for the animals, they are also less effective in deterring theft (American Association of Bovine Practitioners, 2020).

The first aim of this study was to infer the relative influence of pressure for distinctiveness, copying and complexity in the cultural evolution of cattle brands in the US state of Kansas between 1990 and 2016. To do this, we conducted generative inference by fitting an agent-based model (ABM) of cattle brand invention, in which simulated ranchers create new brands under varying levels of copying or pressure for distinctiveness, and pressure for complexity, to the spatiotemporal dynamics of brands and their constituent components. We used a simplified version of the model that we originally preregistered and made no specific hypotheses. The original preregistered analysis and hypotheses can be found in the first version of the preprint,^2^ and a justification for departure from preregistration is included in the SI.

### 1.2. Second aim: time- and space-averaging

The second aim of this study was to use the high temporal and spatial resolution of our data to assess the influence of time- and space-averaging on the predictability of data. Cultural transmission processes are most easily differentiated from one another by their spatiotemporal dynamics (Barrett, 2019; Wilder & Kandler, 2015). Time- and space-averaging, or the mixing of temporal or spatial structure in data (Perreault, 2019), disrupts such dynamics and makes it much more difficult to accurately infer processes from patterns in data (Garvey, 2018). Averaging, also referred to as mixing, is usually an organic result of how artefacts are deposited in the archaeological record, but it is also sometimes imposed by researchers for analytical convenience (Lyman, 2003; Perreault, 2018). In some cases, time averaging may dilute evidence for other processes and increase the fit of data to neutral models (Brantingham, 2003), as empirically observed in fossil assemblages (Tomašových & Kidwell, 2010). However, simulation-based evidence suggests that time-averaging can sometimes make it more difficult to correctly detect random copying depending on the methods used (Madsen, 2012; Porčić, 2015; Premo, 2014). The effects of space-averaging are less well understood, but there is some empirical evidence that time- and space-averaging affects statistical signals in similar ways (Lyman, 2003).

We investigated the effect of time- and space-averaging on signals of copying at the level of brand components (i.e. subunits). In cultural systems where variants are composed of subunits, copying should leave statistical signals analogous to linkage disequilibrium (i.e. genetic alleles being non-randomly associated; Lewontin, 1995) or to pointwise mutual information (i.e. one item carrying information about the presence of another; Shannon, 1951). For example, if components are sometimes copied wholesale, or together in groups, then the co-occurrence of components in brands will be higher than expected by chance (Morin & Miton, 2018). The shuffling model is a modified derivation of pointwise mutual information that predicts the prevalence of variants assuming that their subunits contain no information about one another (Morin & Miton, 2018). In other words, the shuffling model predicts what the prevalence of brands would be if all components in the population were randomly copied proportional to their current frequencies, with no wholesale copying. In their study of heraldic coats of arms, Morin and Miton found that their shuffling model managed to predict the popularity of coats of arms to a substantial degree. They also found that the presence of heraldic coats of arms is better predicted by a random copying model when it is applied to space-averaged data from all of Europe rather than from individual provinces (Morin & Miton, 2018). In this case, space-mixing might disrupt local clusters of design elements that result from wholesale copying of coats of arms within families, thus improving the predictions of a model that assumes random copying of individual design elements.

Do time- and space-mixing boost signals of random copying? To answer this question, we compared the performance of an adapted version of Morin and Miton's shuffling model (Morin & Miton, 2018) on structured, time-mixed and space-mixed datasets. For each of these datasets, we first used the shuffling model to calculate the predicted prevalence of every possible combination of components. Then, we used linear mixed models to compare the accuracy of these predictions after accounting for factors like complexity. We hypothesised that the shuffling model would perform better on the time- and space-mixed datasets compared with structured datasets. The structured datasets built for this goal were also used to test whether brands that are closer to each other in time tend to be more similar.

## 2. Methods

### 2.1 Data

The data for this study come from the Brands Program of the Kansas Department of Agriculture, which has periodically published books containing all registered cattle brands in the state since 1941.^3^ In the early 1990s Kansas’ Brands Program began to use a 13-digit coding system to catalogue all of their cattle brands, which has been used in the seven most recent brand books: 1990, 1998, 2003, 2008, 2014, 2015 and 2016. Kansas’ coding system includes information about brands’ components, angles of rotation and location on the body. Of the brand books that include this coding system, only the latter four are available in original PDF formats that can be accurately transcribed with optical character recognition (OCR). The three earlier books are only available as scans of the printed books, and thus have noise and artefacts that interfere with OCR. We used a custom neural network in Tesseract OCR to automatically extract the cattle brand codes and zip codes from 2008, 2014, 2015 and 2016, and manually transcribed the brand book from 1990 to maximise the temporal depth of our data. 1998 and 2003 were excluded owing to time and labour constraints. The estimated error rate in transcription, based on a manual check of 1500 brands, was 0% for brand codes and 0.4% for locations. Details about the automated and manual transcription can be seen in the data GitHub.^4^

Each brand code has 13 digits, composed of four three-digit component codes and one single-digit location code. The three-digit component codes contain the abbreviation for the component and a number denoting its angle of rotation. Kansas’ coding system has some redundancies, such as ‘\’ and ‘/’ having separate abbreviations despite being rotated versions of the same component. We collapsed and converted redundant abbreviations into single categories with corrected angles of rotation. Kansas’ Brands Program only allows duplicated cattle brands if they are located on different parts of the animal. Duplicated brand codes (as opposed to the cattle brands themselves) can also occur when the same components are combined in a different arrangement. We chose to remove duplicate brand codes from within the same zip code, which usually occur when a family or ranch has registered a single brand multiple times for different locations, but we chose to keep duplicate brand codes from different zip codes, which usually correspond to different arrangements of the same components. In total, we have 81,063 brand codes from 1990 to 2016, comprised 103 unique components (details in the code GitHub^5^).

### 2.2. Generative inference (first aim)

The ABM simulates ranchers creating new brands every year based on the components present in the brands used by ranchers in the entire state, as well as the dual constraint of simplicity/complexity. Prior simulations showed that including angles of rotation in the model yielded results incompatible with the real data, so we chose to exclude them from our analysis (Figure S4).

New brands are created by sampling components from existing brands within the entire state. All of the components are compiled into a frequency table that is used for weighted random sampling. The probability *P*(*x*) that a rancher uses a particular component *x* is based on the frequency of *x* raised to an exponent *C*, normalised against the probability of using other components in the population:
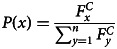


Negative values of *C* cause the rarest components to have the highest probability of adoption, thus leading to more distinctive brands. We operationalise this situation (*C* < 1), where the probability of adoption is negatively related to frequency, as pressure for distinctiveness. *C* = 0 produces completely random brands independent of component frequencies. Positive values of *C* cause the common components to have the highest probability of adoption and introduces redundancy in brand designs. We operationalise this situation (*C* > 1), where the probability of adoption is positively correlated with frequency, as copying. A value of *C* = 1 leads to random (i.e. unbiased) copying, where components are used proportional to their current frequencies. The parameterisation of *C* in this model is directly based on standard models of anticonformity and conformity in cultural transmission (Crema et al., 2014, 2016; Lachlan et al., 2018; Youngblood et al., 2021; Youngblood & Lahti, 2022), and a full comparison can be seen in Figure S3. To simulate pressure for simplicity/complexity, the number of components in each new brand is drawn from a Poisson distribution where *λ* is the parameter of interest (henceforth complexity; see Figure S1 for examples). At lower values of *λ* (e.g. 0.5) one-components brands are much more common, and at higher values of *λ* (e.g. 15) four-components brands are much more common. An intermediate value of *λ* (e.g. 3) leads to an equal probability of two- and three-component brands and a low probability of one- and four- component brands. No new components were added to Kansas’ standardised set during our study period, so we do not simulate the innovation of new component types.

At the beginning of each year in the ABM a set of *N*_new_ brands is created, where *N*_new_ is the average number of new brands that appear each year in the observed data. Each new brand is assigned a zip code, where the probability of each zip code is proportional to its frequency in all years of the observed data. After new brands are created a set of *N*_old_ random brands is removed, where *N*_old_ is the average number of brands that disappear each year in the observed data.

The ABM is initialised with the data from 1990 and runs through every year until 2016. In order to fit the parameters of the ABM to the observed data we calculated the following set of summary statistics from 2008, 2014, 2015 and 2016:

1. proportion of components that are the most common type;
2. proportion of components that are the most rare type;
3. Shannon's diversity index of components;
4. Simpson's diversity index of components;
5. Jaccard index of components (zip codes);
6. Morisita–Horn index of components (zip codes);
7. Jaccard index of components (counties);
8. Morisita–Horn index of components (counties);
9. mean Levenshtein distance between brands (from random 10%).

Note that these summary statistics are calculated using all of the brands that are present in a given year (e.g. 2014 includes all brands created before and during 2014 that have not yet been removed from the population). This is process is conducted separately for the real and simulated data so that the two can be compared. These summary statistics were chosen because they capture the overall diversity of components with an emphasis on both common (1 and 4) and rare (2 and 3) variants, the spatial diversity of components at the level of both zip codes (5 and 6) and counties (7 and 8), and a proxy for the average perceptual distance between brands (9). For all diversity metrics we calculated their Hill number counterparts, because they are measured on the same scale (Roswell et al., 2021) and better account for relative abundance (Chao et al., 2014). Shannon's diversity index emphasises rarer types whereas Simpson's diversity index emphasises more common types. The Jaccard and Morisita–Horn indices were similarly chosen for their complementarity. The Morisita–Horn index is a commonly used abundance-based beta diversity index (Chao et al., 2006), whereas the Jaccard index is the most robust of the incidence-based beta diversity indices to sampling error (Schroeder & Jenkins, 2018). We calculated beta diversity at both the zip code and county level to assess spatial diversity at two different resolutions. The mean Levenshtein distance (a measure of edit distance), or the minimum number of insertions, deletions and substitutions required to convert one sequence to another, was calculated from a random 10% of brands.

The same summary statistics were calculated from the real data from 2008, 2014, 2015 and 2016. Then, the random forest version of approximate Bayesian computation (ABC) was conducted using the *abcrf* package in R (Raynal et al., 2019), with the following steps:

1. 500,000 iterations of the ABM were run to generate simulated summary statistics for different values of *λ* and *C*.
2. The output of these simulations were combined into a reference table with the simulated summary statistics as predictor variables, and the parameter values as outcome variables. *λ* was log-transformed because it is non-negative, as recommended for non-linear regression-based ABC (Blum & François, 2010; Sisson et al., 2018).
3. A random forest of 1000 regression trees was constructed for each parameter using bootstrap samples of 80% of the data from the reference table. Random forest parameters were tuned using a random 10% of the data with the *tuneRanger* package in R (*λ*, split features = 6 and minimum node size = 3; *C*, split features = 17 and minimum node size = 3) (Probst et al., 2018).
4. Each trained random forest was provided with the observed summary statistics, and each regression tree was used to generate posterior distributions for both parameters.

Calculating all nine summary statistics for each of the four years means that we conducted ABC with 36 summary statistics in total. Random forest ABC is robust to the number of summary statistics (Pudlo et al., 2016) so we did not have to reduce the dimensionality prior to inference (Blum et al., 2013). As an additional robustness check, we fit the agent-based model to simulated data from two combinations of known parameter values: (1) *λ* = 3 and *C* = −1 (intermediate complexity and pressure for distinctiveness); and (2) *λ* = 3 and *C* = 1 (intermediate complexity and random copying). In both cases, the random forest ABC recovered the correct input parameters (Figures S6 and S7). Visualisations of model output for the SI were conducted with principal component analysis and uniform manifold approximation and projection (McInnes et al., 2018).

*C* was assigned a normal prior distribution with a mean of 0 and standard deviation of 2. *λ* was assigned a gamma prior distribution with a shape parameter of 0.9 and a rate parameter of 0.2. This distribution was chosen because, based on 200,000 samples, it includes a relatively even spread across the component probabilities shown in Figure S1 while allowing for some extreme values (minimum of ~0, maximum of 75).

### 2.3. Shuffling model (second aim)

The shuffling model takes a set of brands and calculates a predicted prevalence for every possible combination of the components within those brands. When it performs well, the shuffling model assigns a higher predicted prevalence to brands that actually exist in the data. We predict that this will be more true for time- and space-mixed datasets than for the original, structured datasets. According to the shuffling model, the predicted prevalence of a brand is simply the product of the frequency of its components in a specific subset of data. For example, the predicted prevalence (*S*) of a brand with three components *A*, *B* and *C* would be:
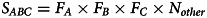
where *F_A_* is the frequency of *A* among brands that do not include *B* or *C*, *F_B_* is the frequency of *B* among brands that do not include *A* or *C*, *F_C_* is the frequency of *C* among brands that do not include *A* or *B* and *N*_other_ is the total number of brands excluding the focal brand.

To apply this model, we first needed to generate structured and mixed datasets. The structured datasets were split into groups according to time, space and complexity. For time, brands were grouped into two age groups: the ‘old’ group (O) included all brands that appear in the 1990 brand book, and the ‘young’ group (Y) included all brands that appear in later brand books. For space, brands were grouped into four rectangular areas of roughly equal size following county borders: northwest (NW), southwest (SW), northeast (NE) and southeast (SE) (see Figure S2). For complexity, brands were grouped into two categories: two-component and three-component brands. One-component brands were excluded because they lack the combinatorics required for the shuffling model, and four-component brands were excluded because they are relatively rare in the dataset (~2%). This structure was used to split the data into 16 subsets, each of which represents a unique combination of time, space and complexity (e.g. O-NE-2 is old northeastern brands with two components).

Once we had structured datasets we generated time- and space-mixed datasets to test our hypotheses about the effects of time- and space- averaging. Each mixed dataset has as many brands as the structured dataset that it imitates. For time-mixed datasets, brands were randomly drawn from the same area independently of time. The time-mixed O-NE-2 dataset was constructed by randomly sampling *n* two-component brands from northeast Kansas across all time periods, where *n* is the number of brands in the structured subset. For space-mixed datasets, brands were randomly drawn from the same time dataset independently of space. The space-mixed O-NE-2 dataset was constructed by randomly sampling old two-component brands from all of Kansas.

For each structured and mixed dataset, we inventoried all of the components present in the brands, listed all of their possible combinations, and used the shuffling model to assign a predicted prevalence to each combination. For each possible combination we noted whether it was actually present in the dataset or not.

To compare the accuracy of the shuffling model when applied to the structured and mixed datasets, we built two linear mixed effects models with predicted prevalence as the outcome variable: one with the structured and time-mixed datasets, and the other with the structured and space-mixed datasets. Whether the dataset was mixed (MIXED: 0 = N, Y = 1), whether the combination of components was actually present in the data (ACTUAL: 0 = N, Y = 1) and complexity (COMPLEXITY: 2 = 0, 3 = 1) were potential fixed effects. Space, time and the specific combination of components were potential varying intercepts. The shuffling model assigns a predicted prevalence of 0 when a component occurs multiple times in a target brand or only occurs in a single brand in the dataset, so all combinations with a predicted prevalence of 0 were dropped from the modelling. The remaining predicted prevalence values fit a lognormal distribution so we log-transformed them prior to modelling. Model choice was conducted using Akaike's information criterion (AIC) calculated from frequentist models fit with the *lme4* package in R (Bates et al., 2015). The best fitting versions of each model were run as Bayesian models in Stan using the *brms* package in R (Bürkner, 2017), using mean field approximation with 5000 samples from 50,000 iterations.

Our first prediction was that ACTUAL = 1 would have a positive effect on the predicted prevalence of a combination of components, which would indicate that the shuffling model works better than chance. Our second prediction was that there would be an informative and significantly positive interaction term ACTUAL*MIXED, or in other words that the predicted prevalence differential between ACTUAL = 1 and ACTUAL = 0 is more important when MIXED = 1. This would indicate that the performance of the shuffling model is higher in mixed datasets. Finally, complexity was included as a control variable because three-component brands have an additional multiplied proportion that probably reduces their prevalence values.

### 2.4. Temporal distance (supplemental analysis)

The structured datasets used for the shuffling model were also used to test whether brands that are closer to each other in time tend to be more similar. First, we pooled together both the old and young brands from each spatial subset (e.g. O-NE-2 + Y-NE-2 = NE-2) and calculated the Levenshtein distance (LD) between each pair of brands. As a reminder, old brands are those that appear in the 1990 brand book and young brands are those that appear in later brand books but not in the 1990 issue. Then, we ran a linear mixed effects model with LD as the outcome variable, whether the brands are both old (OO), both young (YY) or one of each (OY) as the predictor variable, and the identities of the compared brands as random effects (i.e. one intercept for the first brand and one for the second). We predicted that pairs of brands that are OY would have a higher LD relative to OO and YY, or, in other words, that brands from different time periods would be more distinct from one another.

## 3. Results

### 3.1. Generative inference (first aim)

**Figure 2.**
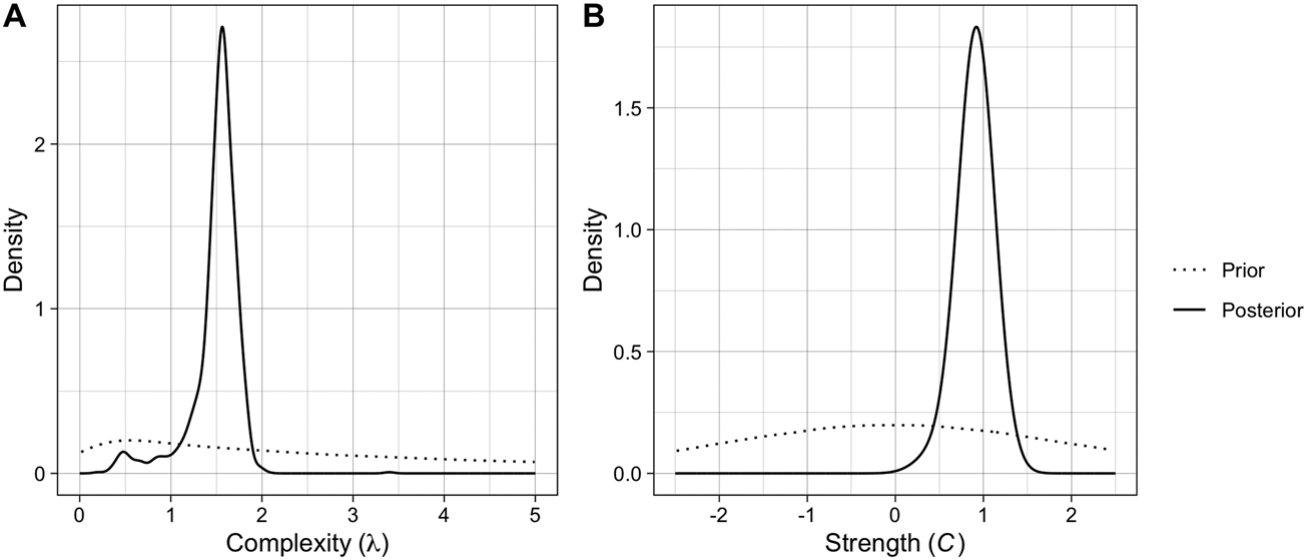
The posterior distributions for the two parameters of the ABM computed with random forest ABC, plotted against the priors (dotted).

The posterior distributions and estimates of the two parameters can be seen in Figure 2 and Table 1, respectively. The posterior for complexity (*λ*) has a median of 1.55 and a relatively tight 95% confidence interval (CI), suggesting that the data are consistent with pressure for intermediate complexity. As a reminder, *λ* controls the shape of the Poisson distribution from which the number of components in new brands is drawn, so a value of 1.55 gives two components a slightly higher probability than three components (Figure S1). The posterior for copying strength (*C*) has a median of 0.92 with a relatively tight 95% CI, suggesting that the data is consistent with copying rather than pressure for distinctiveness, at a level slightly below random copying (*C* = 1). Posterior simulations demonstrate that the fitted parameter values produce output close to the observed data (Figure S5).

**Table 1.** The median estimates, 95% quantile-based credible intervals, and root mean square log/logit-transformed errors for each parameter. *λ* controls the shape of the Poisson distribution from which the number of components in new brands is drawn, and *C* is the exponent to which the components frequencies are raised.

	M	95% CI	RMSLE
*λ*: complexity	1.55	[0.56, 1.82]	0.13
*C*: strength	0.92	[0.44, 0.96]	0.25

### 3.2. Shuffling model (second aim)

Brand appears to be the only grouping variable that explains a high level of variance in the data (time-mixed, ICC_brand_ = 0.926, ICC_space_ = 0.021, ICC_time_ = 0.009; space-mixed, ICC_brand_ = 0.923, ICC_space_ = 0.003, ICC_time_ = 0.002), so we included it as a varying intercept in both models. Adding complexity as a control variable improved model fit (time-mixed, ΔAIC = 5120; space-mixed, ΔAIC = 4852). Adding ACTUAL as a fixed effect improved model fit (time-mixed, ΔAIC = 1377; space-mixed: ΔAIC = 2388) and adding ACTUAL*MIXED as an interaction further improved model fit (time-mixed, ΔAIC = 23; space-time, ΔAIC = 403). The best fitting models for both the time-mixed and space-mixed datasets had the following specification:
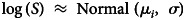

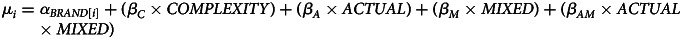

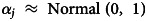

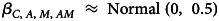

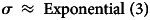


The results of the best fitting models can be seen in Table 2.

**Table 2.** The mean estimates and 95% credible intervals for each parameter in the best fitting models for the time-mixed (left) and space-mixed (right) subsets

	Time-mixed		Space-mixed	
	Estimate	95% CI	Estimate	95% CI
*α*: intercept	0.75	[0.75, 0.76]	0.74	[0.74, 0.74]
*β_C_*: complexity	−1.28	[−1.28, −1.28]	−1.21	[−1.21, −1.21]
*β_A_*: actual	0.13	[0.12, 0.13]	0.16	[0.15, 0.16]
*β_M_*: mixed	−0.007	[−0.009, −0.005]	−0.02	[−0.02, −0.01]
*β_AM_*: actual*mixed	0.02	[0.02, 0.03]	0.05	[0.04, 0.06]
*σ*: standard deviation	0.29	[0.29, 0.29]	0.29	[0.29, 0.29]

**Figure 3.**
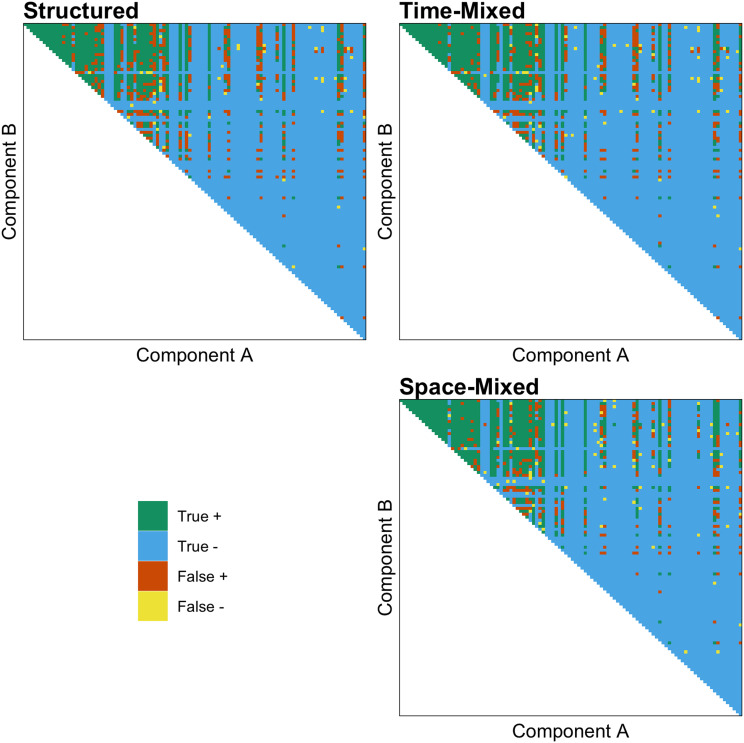
The accuracy of the shuffling model's predictions when applied to a structured (left panel), time-mixed and space-mixed (right panel) version of Y-SE-2, the dataset with the highest sample size. Each point above the diagonal is a unique combination of components, and components on both axes are ranked by their commonness in the entire dataset. Green is a true positive (exists and *S* ≥ 1), blue is a true negative (does not exist and *S* < 1), orange is a false positive (does not exist and *S* ≥ 1), and yellow is a false negative (exists and S < 1).

As predicted, we found that both the effect of ACTUAL and the interaction between ACTUAL and MIXED are positive, indicating that the shuffling model works better than chance and performs better in both time- and space-mixed datasets. Figure 3 demonstrates the accuracy of the shuffling model's predictions when applied to a structured, time-mixed and space-mixed dataset (i.e. Y-SE-2, which has the highest sample size). The effect is subtle, but when applied to mixed datasets (right panel) the shuffling model has more true and fewer false positives, apparently because more of the possible combinations of common components are represented.

We found that complexity has the expected negative effect on predicted prevalence. The *R*-hat values are all equal to 1, and the effective sample sizes are all greater than 1000, showing that both models have converged. The results of a frequentist version of the best fitting model can be seen in Table S2.

As a follow-up, we repeated our generative inference analysis after randomly mixing the years and locations of the real brands (not preregistered) and found that the detected signals of copying were robust to time- and space-mixing (Figure S8).

### 3.3. Temporal distance (supplementary aim)

The temporal distance model had the following specification:
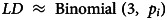

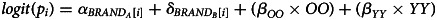

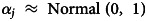

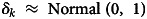

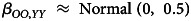


The results of the temporal distance model can be seen in Table 3. As predicted, we found that the effect of compared brands being from the same time period (e.g. OO or YY) on their Levenshtein distance was negative. In other words, we found that brands from different time periods are more distinct from one another. The *R*-hat values are all equal to 1, and the effective sample sizes are all greater than 1000, showing that the model has converged.

**Table 3.** The mean estimates and 95% credible intervals for each parameter in the best fitting model.

	Estimate	95% CI
α*_j_*: intercept a	0.60	[0.59, 0.60]
*δ_k_*: intercept b	1.05	[1.05, 1.05]
*β_OO_*: both old	−0.20	[−0.20, −0.19]
*β_YY_*: both young	−0.30	[−0.30, −0.30]

## 4. Discussion

Based on the results of generative inference, the spatiotemporal dynamics of Kansas cattle brands between 1990 and 2016 are consistent with copying of components across the entire state. Ranchers do not maximise the distinctiveness of their brands beyond the basic constraint of uniqueness, but they do appear to create brands of intermediate complexity. In support of our hypothesis, we found that time- and space-averaging improves the ability of the shuffling model, which assumes random copying at the level of components, to predict the presence and absence of brands in our data. This finding is supported by the fact that our generative inference results remain consistent with copying after the years and locations of brands have been shuffled. Finally, brands from the same time period are more similar to one another than brands from different time periods, as would be expected when copying is present.

Our finding that signals of copying are robust to, and possibly boosted by, time- and space-averaging is significant in light of the previous literature on this topic. Several simulation studies have found that time-averaging makes it more difficult to correctly detect random copying using classic neutrality tests like Slatkin's exact test (Madsen, 2012; Porčić, 2015; Premo, 2014). Such tests are very sensitive to evenness, and thus the long tail of distributions (Hill, 1997) and departures from neutrality induced by time-averaging appear to be due to the overrepresentation of rare and fleeting variants (Premo, 2014). Previous simulation studies have assumed that an infinite number of new variants are possible, though, and time-averaging may not have this effect in systems like cattle brands where variation is bounded because new variants are produced from a finite set of constituent parts. Additionally, Premo (2014) found that if the entire frequency distribution of variants is considered, then signals of random copying appear to be robust to time-averaging (or even slightly boosted by it, even at low sample sizes). This is consistent with our findings, as well as empirical evidence that time-averaging boosts signals of neutral evolution in fossil assemblages (Tomašových & Kidwell, 2010).

**Figure 4.**
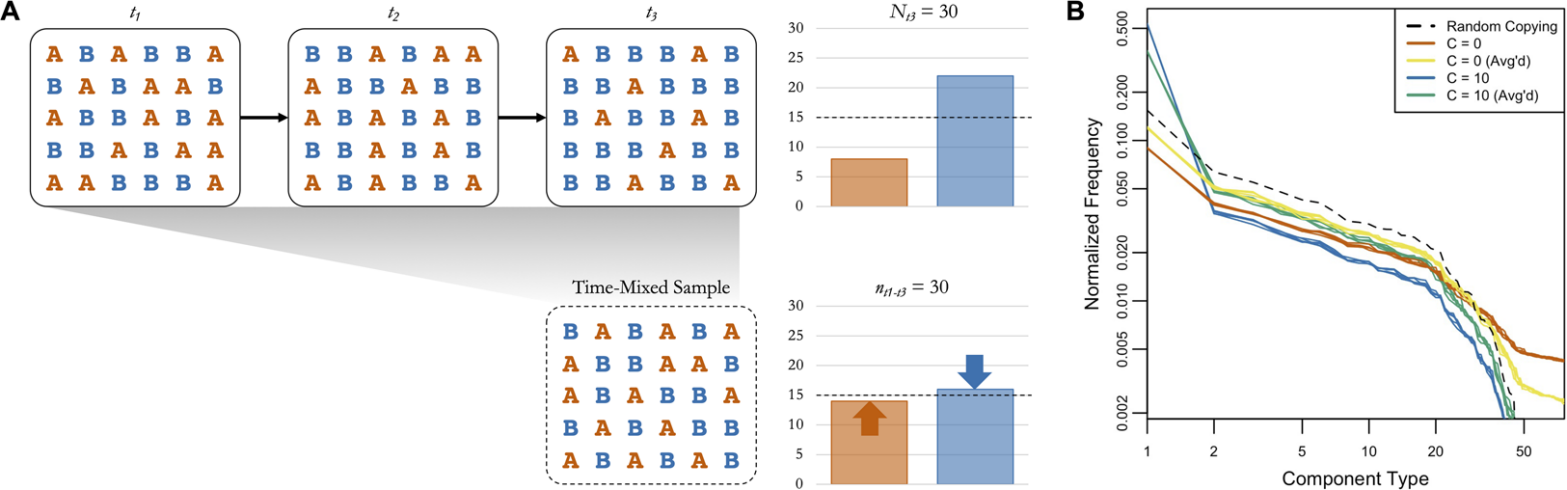
(a) A basic simulation of cultural transmission over three timesteps, with a fully-connected population of 30 agents transmitting two variants (A and B) with a conformity bias (*C* = 2). A and B are equally represented in the first timestep. The bar plot in the top right shows the frequencies of A and B in *t*_3_, and the bar plot in the top left shows the frequencies of A and B in a time-mixed subsample of the same size from *t*_1_ to *t*_3_. The dashed lines show the expected frequencies under random copying. (b) The simulated component frequency distributions from the cattle brand ABM under several conditions (five iterations each). Orange and blue are the frequency distributions from 2016 when *C* = 0 (frequency information does not matter) and *C* = 10 (extreme conformity), respectively. Yellow and green are the frequency distributions from time-mixed subsamples of the same size collected across all years when *C* = 0 and *C* = 10, respectively. The black dashed line is the frequency distribution expected when *C* = 1 (random copying).

Figure 4 provides an example of the logic of averaging boosting signals of random copying, with a basic simulation of cultural transmission over three timesteps. In *t*_1_ the two cultural variants A and B are equally represented in the population. In *t*_2_ and *t*_3_ a conformity bias (*C* = 2) causes B to increase in frequency, as shown in the plot of the full population in *t*_3_ in the top right. If we only have access to a subsample from *t*_1_ to *t*_3_, though, the frequency of the rare variant (A) is higher and the frequency of the common variant (B) is lower (see bottom right), bringing them closer to the expectation under random copying (dashed line). If we simulate this with our cattle brand ABM, using the same starting point and temporal dynamics as our main analysis, we see a similar pattern (Figure 4). The orange and blue lines show the simulated frequency distributions of components in 2016 when *C* = 0 (frequency information does not matter) and when *C* = 10 (extreme conformity). If we take random subsamples of the same size from all years, shown in yellow and green, it brings the frequency distributions closer to the expectation under random copying, shown in black. When *C* = 0 the time-mixing boosts the frequencies of common components at the expense of rare ones, and when *C* = 10 it boosts the frequencies of rare components at the expense of common ones.

There are several important differences between cattle brands and typical archaeological datasets that should be highlighted. First, the relative time-scale of averaging in our study is much smaller than that typically seen in archaeological assemblages. The effects of time-averaging generally increase with the aggregation window (Perreault, 2018; Wilder & Kandler, 2015), with the onset of effects occurring when the window is about the same as the average lifetime of variants (Madsen, 2012). Cattle brands are often inherited within families and can have relatively long use-lives, and we suspect that the ~25-year window of our study is close to the average lifetime of a cattle brand. Our study shows that time-averaging can have a significant impact on the detectability of copying with a much narrower time window than previously assumed. Second, time-averaging has bigger effects when innovation rates are high (Madsen, 2012), and no new component types were innovated during the course of our study period. Combined with the relatively small averaging window, we think that this explains the subtlety of the observed effects of time- and space-averaging (see Table 2 and Figure 3). Finally, the time- and space-averaging in our study occurred at the level of brands, while copying and pressure for distinctiveness were modelled at the level of components. In previous studies, models have assumed that cultural variants are copied discretely as types at the same level of analysis at which time- and space-averaging occurs (Madsen, 2012; Porčić, 2015; Premo, 2014). If we use pottery traditions as an example, a ‘type’ is actually a complex combination of different techniques or design elements. Copying probably occurs at the level of these techniques and elements (or combinations of them), whereas time- and space-averaging occur at the level of the artefacts. We think that continuous cultural variation may contain additional information that is affected differently by averaging or missing data (Premo, 2021).

## Data Availability

The preregistration document is available on OSF (https://osf.io/d4qv3), the data and transcription methodology are available on our data GitHub (https://github.com/masonyoungblood/cattle_brand_data), and the agent-based model is available on our code GitHub (https://github.com/masonyoungblood/CattleBrandABM).

## Footnotes

1
https://agriculture.ks.gov/divisions-programs/division-of-animal-health/brands-program

2
https://psyarxiv.com/h5t7b/

3
https://cdm16884.contentdm.oclc.org/digital/collection/p16884coll5/id/317/rec/2

4
https://github.com/masonyoungblood/cattle_brand_data

5
https://github.com/masonyoungblood/CattleBrandABM

## Supplementary material

For supplementary material accompanying this paper visit https://doi.org/10.1017/ehs.2023.5. click here to view supplementary material
