# Supplementary material for: Statistical signals of copying are robust to time- and space-averaging
Source: Evol Hum Sci. 2023 Apr 13;5:e10. doi: 10.1017/ehs.2023.5 (PMC10426036; doi:10.1017/ehs.2023.5)
Supplement: Supplementary file 1 [file S2513843X23000051sup001.pdf]

Supplementary Information

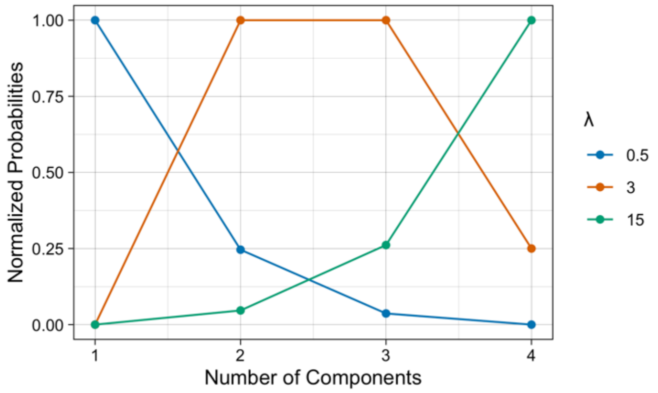

Figure S1. The normalized probabilities of creating a brand with between one and four components when *λ* is 0.5, 3, and 15. Note that the normalization here is only for plotting purposes.

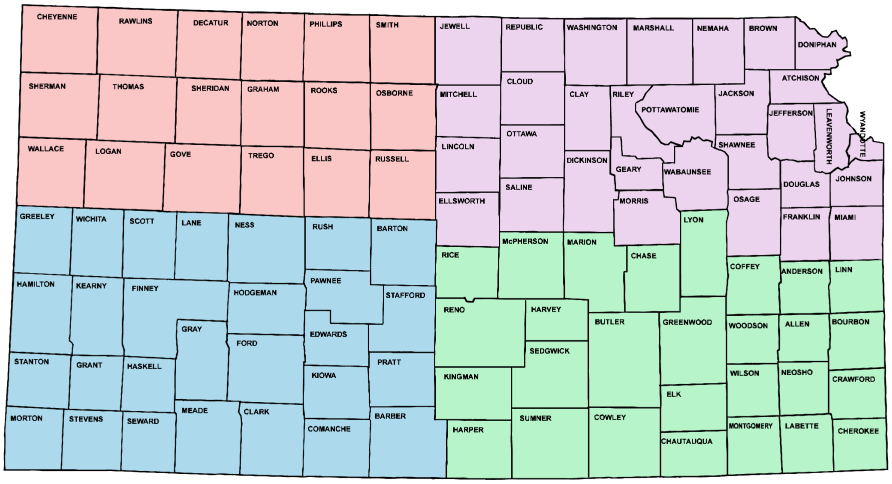

Figure S2. A map of Kansas split into the four rectangular areas of approximately equal size that was used for the structured and mixed datasets: NW in red, SW in blue, NE in purple, SE in green.

	**NE**	**NW**	**SE**	**SW**
**O-2**	3,719	3,255	4,603	5,027
**O-3**	979	800	1,429	1,605
**Y-2**	9,866	8,765	12,207	11,280
**Y-3**	3,565	2,586	4,839	4,132

Table S1. The sample sizes for each of the 16 subsets (e.g. O-NE-2 has 3,719 brands).

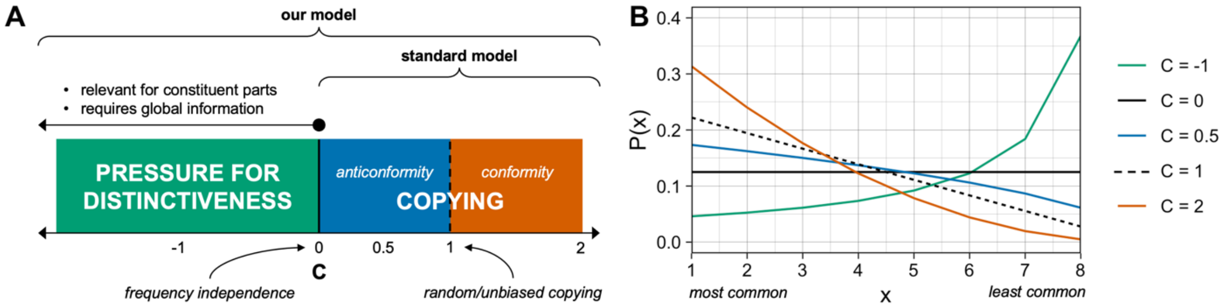

Figure S3. A comparison of the model used in this study with standard models of anticonformity and conformity in cultural transmission, both of which exponentiate the frequency of variants by a parameter (henceforth *C* for comparison). Panel A shows the processes that different values of C correspond to, and panel B shows the effect that these values have on the probability of adoption of cultural variants. In panel B, the frequency distribution of the eight variants is linear from 8 to 1—F(1) = 8, F(2) = 7, etc. In the standard model, agents are only able to adopt the variants of the demonstrators that they interact with. When $0 < C < 1$ you get anticonformity: rare variants are more likely to be adopted than expected under random/unbiased copying (comparing blue line to dotted black line in panel B). Likewise, when $C > 1$ you get conformity: common variants are more likely to be adopted than expected under random/unbiased copying (comparing orange line to dotted black line in panel B). Researchers using these standard models do not explore $C \leq 0$ (Crema et al., 2014, 2016; Lachlan et al., 2018; Youngblood et al., 2021; Youngblood & Lahti, 2022), because it is not usually feasible for the rarest variants to have the highest probability of adoption if the vast majority of agents will not encounter them (in fact, they often set a lower bound of 0.5; Crema et al., 2014, 2016). In our model, though, agents create brands from a standardized list of components allowed by the state of Kansas, and no new types are innovated over the course of the study. This global information makes it possible for the use of components to be decoupled from ($C = 0$) or inversely related to ($C < 0$) their current frequencies. For the purposes of this study, we consider this "pressure for distinctiveness", where the probability of adoption is negatively related to frequency, to be qualitatively different from what is usually referred to as "copying", where the probability of adoption is positively related to frequency. This interpretation may only be relevant to the invention of cultural traits produced from a bounded set of constituent parts. For example, in a standard cultural transmission model $C < 0$ could be interpreted as *extreme* anti-conformist copying, but the important point for us is that this extreme bias at the level of components leads to increased distinctiveness at the level of brands.

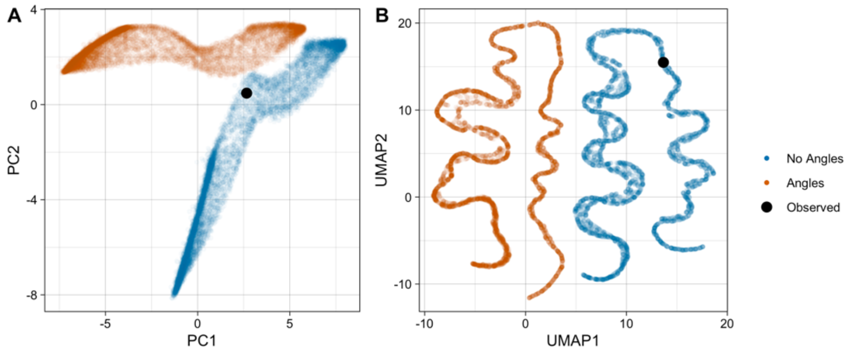

Figure S3. The results of 10,000 prior simulations run with (orange) and without (blue) considering angles, alongside the observed summary statistics (black). Panel A shows the first two principal components from a PCA, and panel B shows the two dimensions from a UMAP run with 15 nearest neighbors and a minimum distance of 0.1. The model with angles clearly does not capture the patterns in the observed summary statistics.

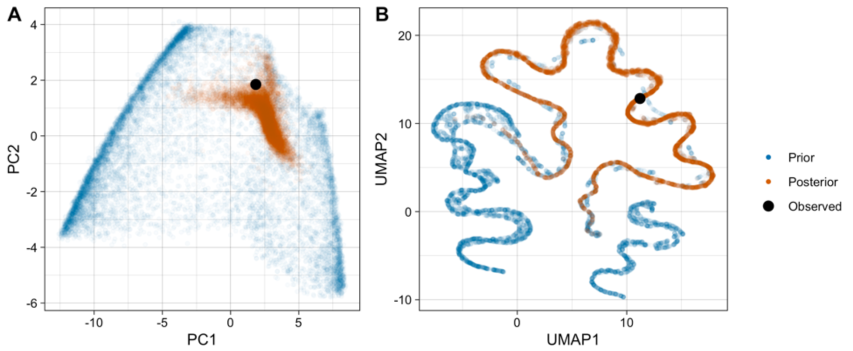

Figure S4. The results of 10,000 prior simulations (orange) and posterior simulations (blue), alongside the observed summary statistics (black). Panel A shows the first two principal components from a PCA, and panel B shows the two dimensions from a UMAP run with 15 nearest neighbors and a minimum distance of 0.1. The simulations from the posterior capture a much smaller portion of the parameter space around the observed data.

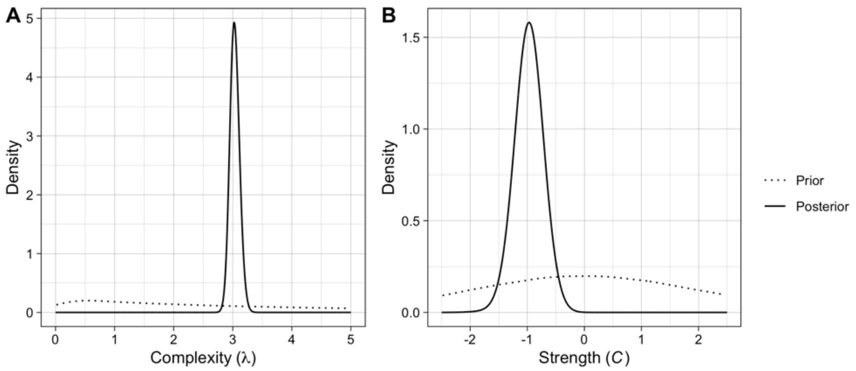

Figure S5. Robustness check: the posterior distributions for the two parameters of the ABM computed with random forest ABC, plotted against the priors (dotted). The ABC was fitted to output of the ABM produced by known parameters: $\lambda = 3$ and $C = -1$.

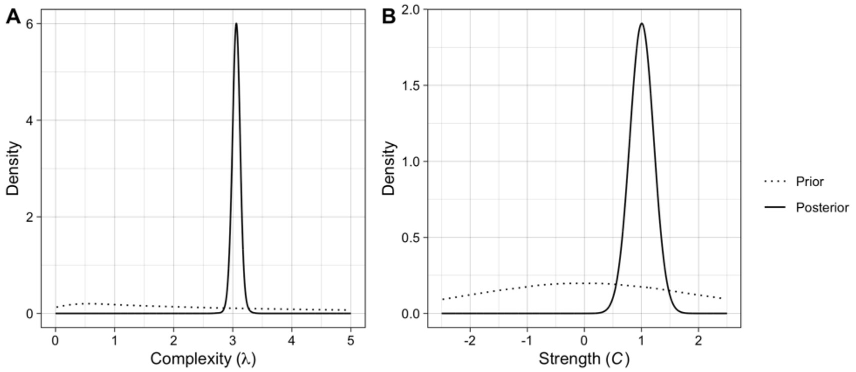

Figure S6. Robustness check: the posterior distributions for the two parameters of the ABM computed with random forest ABC, plotted against the priors (dotted). The ABC was fitted to output of the ABM produced by known parameters: $\lambda = 3$ and $C = 1$.

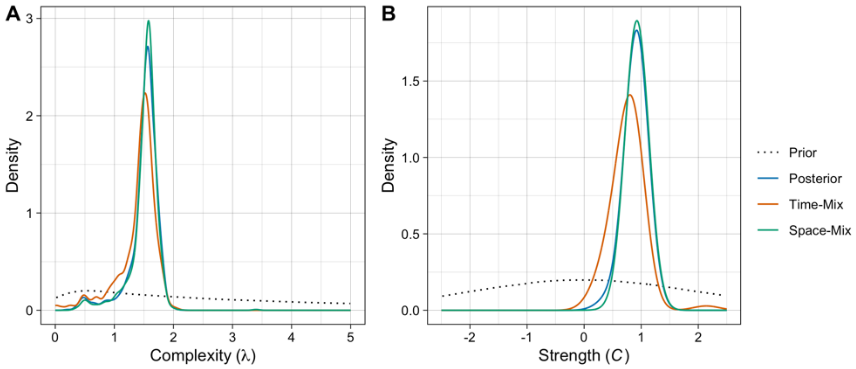

Figure S7. The posterior distributions for the two parameters of the ABM computed with random forest ABC when the data are structured (blue), time-mixed (orange), and space-mixed (green), plotted against the priors (dotted). The posterior for both parameters remains roughly the same across the three conditions.

	Time-Mixed			Space-Mixed		
	**Estimate**	**SE**	**95% CI**	**Estimate**	**SE**	**95% CI**
α: intercept	0.75	0.017	[0.73, 0.78]	0.73	0.017	[0.68, 0.76]
β_C: complexity	-1.28	0.018	[-1.31, -1.25]	-1.21	0.017	[-1.24, -1.15]
β_A: actual	0.12	0.004	[0.11, 0.14]	0.15	0.004	[0.14, 0.16]
β_M: mixed	-0.005	0.0009	[-0.007, -0.003]	-0.02	0.0009	[-0.02 0.02]
β_{AM}: actual*mixed	0.02	0.004	[0.01, 0.03]	0.05	0.004	[0.04, 0.06]
σ: std deviation	0.29	NA	[0.29, 0.29]	0.29	NA	[0.29, 0.29]

Table S2. The estimates, Wald standard errors, and 95% confidence intervals (bootstrapped from 50 simulations) from frequentist versions of the best fitting models for the time-mixed (left) and space-mixed (right) datasets. The standard error for σ is omitted because Wald standard errors are generally considered to be poor summaries of the uncertainty of variance components.

Departures from preregistration

The original version of the agent-based model included a more complex model with separate parameters for copying and distinctiveness within defined geographic radii. During peer review, we discovered that random forest ABC was unable to recover known parameter values from simulated data using this model, so we have made the following simplifications to the generative inference portion of the analysis:

1. The two geographic radii were fixed to the maximum pairwise distance between zip codes in Kansas. This means that pressure for copying and distinctiveness are now global—across the entire dataset—rather than having a spatial pattern.
2. The separate parameters C and D were combined into a single parameter C, for which positive values produce copying and negative values produce distinctiveness. We compressed the two parameters because C and D counteract each other when the geographic radii have the same value (e.g. $C = 1$ and $D = -1$ in the original model would be equivalent to $C = 0$ in the new model). The prior for C was modified to be symmetrical around zero.
3. We originally hypothesized that there would be pressure for distinctiveness within a smaller radius, and some level of copying within a larger radius. This prediction was logically consistent with the shuffling model, which assumes copying across the entire state, because the pressure for distinctiveness would be geographically bounded. In the new global model, where the spatial pattern has been removed, a prediction of distinctiveness is no longer logically consistent with the shuffling model. As such, we have opted to remove our original hypotheses from the new version of the manuscript.
